# Novel Platforms for the Development of a Universal Influenza Vaccine

**DOI:** 10.3389/fimmu.2018.00600

**Published:** 2018-03-23

**Authors:** Arun Kumar, Trine Sundebo Meldgaard, Sylvie Bertholet

**Affiliations:** ^1^GSK, Research and Development Center, Siena, Italy; ^2^Linköping University, Linköping, Sweden; ^3^DTU Nanotech, Technical University of Denmark, Copenhagen, Denmark; ^4^GSK, Research and Development Center, Rockville, MD, United States

**Keywords:** influenza, hemagglutinin, virus-like particles, universal flu vaccine, neutralizing antibodies, vaccination strategies, functional antibody responses

## Abstract

Despite advancements in immunotherapeutic approaches, influenza continues to cause severe illness, particularly among immunocompromised individuals, young children, and elderly adults. Vaccination is the most effective way to reduce rates of morbidity and mortality caused by influenza viruses. Frequent genetic shift and drift among influenza-virus strains with the resultant disparity between circulating and vaccine virus strains limits the effectiveness of the available conventional influenza vaccines. One approach to overcome this limitation is to develop a universal influenza vaccine that could provide protection against all subtypes of influenza viruses. Moreover, the development of a novel or improved universal influenza vaccines may be greatly facilitated by new technologies including virus-like particles, T-cell-inducing peptides and recombinant proteins, synthetic viruses, broadly neutralizing antibodies, and nucleic acid-based vaccines. This review discusses recent scientific advances in the development of next-generation universal influenza vaccines.

## Introduction

Seasonal influenza viruses circulate worldwide, spread easily from person to person, and result in the hospitalization of three to five million individuals worldwide each year (1, 2). These infections are responsible for 250,000–500,000 deaths, mainly among those with immature or compromised immunity, e.g., young children, elderly adults, and critically ill patients (2). However, all age groups can be affected, and the impact can increase significantly with an emergent human influenza-virus strain during a pandemic (3). Influenza viruses are constantly evolving through genome mutation and reassortment. During the past 100 years, new emergent influenza-virus strains have regularly appeared in human populations (“Spanish flu” in 1918 caused by the H1N1 subtype, “Asian flu” in 1957 by H2N2, “Hong Kong flu” in 1968 by H3N2, “Russian flu” in 1977 by H1N1, and “swine flu” in 2009 by H1N1). In addition, limited outbreaks of avian influenza strains in humans threaten the evolution of one or more of these viruses to the point of sustained human-to-human transmission, for example, H5N1 (“bird flu”), H7N9, H5N6 as well as virus variants transmitted from pigs to human (H1N1v, H1N2v, and H3N2v) (4–8). Pandemic influenza has claimed millions of lives globally; the 1918–1920 H1N1 pandemic alone claimed 50–100 million lives (4, 9).

The genome of the influenza virus consists of 8 single-stranded RNA segments encoding 11 proteins, including the surface glycoproteins hemagglutinin (HA) and neuraminidase (NA). The human influenza virus is classified into three distinct types A, B, and C, on the basis of major antigenic differences. Influenza A and B viruses are responsible for annual human epidemics, whereas the influenza C virus is known to infect both humans and pigs and causes very mild upper respiratory tract disease in humans (10, 11). The influenza A virus is classified into 18 HA (H1–H18) and 11 NA (N1–N11) subtypes on the basis of HA and NA glycoproteins (3, 9, 12). On the basis of antigenic properties and structural features, influenza A virus HA subtypes can be further classified into groups 1 (H1, H2, H5, H6, H8, H9, H11, H12, H13, H16, H17, and H18) and 2 (H3, H4, H7, H10, H14, and H15), which comprise different clades (A clade is a group of influenza viruses share homologous features and evolved from a common ancestor) (13, 14). Only a single subtype of influenza B virus has been identified, and this subtype comprises two distinct antigenic lineages (B/Victoria and B/Yamagata) that diverged more than 40 years ago and co-circulate at variable levels in humans (3, 15).

Vaccination is an effective approach for the control and prevention of influenza. Currently, trivalent inactivated-virus (TIV) vaccines against seasonal influenza viruses are the most frequently used influenza vaccines with a steady migration to tetravalent or quadrivalent vaccines (QIV). TIV vaccines are composed of three influenza-virus strains (2 A subtypes, H3N2, H1N1, and 1 B type) selected primarily on the basis of forecasted prevalence during the targeted influenza season. QIV vaccines include the second B lineage (16). TIV vaccines come in three different formulations; the whole virus, split virus, and subunit. Whole-virus vaccines are prepared from embryonated chicken eggs, inoculated with virus, followed by chemical inactivation and purification steps. Split-virus vaccines are prepared by treatment of influenza-virus particles by diethyl ether or detergent (e.g., ammonium deoxycholate), which dissociates the viral lipid envelope exposing all viral proteins (17, 18). Subunit vaccines contain HA and NA proteins and are prepared by applying further purification steps with detergents or diethyl ether (19, 20). A recombinant HA (rHA)-based subunit vaccine has been approved recently; and this vaccine showed higher seroconversion rates in healthy adults including elderly adults, compared with a non-recombinant TIV vaccine (21).

Inactivated vaccines primarily induce protective antibodies against epitopes on HA. Split and subunit formulations are used more frequently than the other formulations and both induce comparable immunity. Whole-virus formulation has been less preferred because of a potential association with increased reactogenicity (22). In the USA, inactivated vaccines are approved from 6 months of age, and rHA formulations are approved for those 18 years of age and older.

Live-attenuated influenza vaccines (LAIV) mimic aspects of natural influenza-virus infection, but without virus pathogenicity, and induce both humoral and cell-mediated immunity (23). Intranasal administration of LAIV induces local mucosal immunity (24) by inducing the secretion of IgA. LAIV is approved in the USA for use in children and adults between 2 and 49 years old (23). However, recently, the Center for Disease Control and Prevention (CDC) Advisory Committee on Immunization Practices (ACIP) recommended that LAIV should not be used during 2017–2018 flu season (25). The ACIP committee made this recommendation because of the lower efficacy of LAIV against A(H1N1)pdm09 viruses during the 2013–2014 and 2015–2016 seasons. Small and Cronin (26) argued that the decision of ACIP was based on Test Negative Case Controlled (TNCC) study design which measures direct rather than indirect protection and just an observational approach (25, 27). Potential causes of reduced effectiveness of A(H1N1)pdm09 in LAIV are evidenced by lower replication in alveolar cell lines and reduced binding to sialic acid receptors (receptors for influenza-virus binding) (28).

The benefits of QIV over TIV are encouraging, and an increasing proportion of influenza vaccination programs are using QIV (29). Nevertheless, both TIV and QIV vaccines require reformulation with new influenza strains each influenza season, and their effectiveness is not guaranteed because it is primarily dependent on the match between the forecasted strains in the vaccines and the actual prevalent strains in circulation.

## Is there a need for a Universal Influenza Vaccine?

The leading challenge in influenza prevention and treatment is identifying effective strategies to deal with the rapidity in which the virus can evolve to evade its host’s immune system or develop resistance to drug treatments. Influenza-virus evolution is typically considered in terms of antigenic drift—the occurrence of minor changes in the virus genotype within the same virus type—and antigenic shift—emergence of new and potentially pandemic strains by the reassortment of viral genomes (13, 30, 31).

Seasonal antigenic drifts usually consist of minor amino acid mutations in the HA globular head domain that may result in some changes to the pattern of glycosylation in this domain (32, 33). These changes in the HA-glycosylation pattern affect the infectivity of the virus and its ability to escape from antibodies elicited by previous strains or vaccines (33). As a result, current influenza vaccines, which provide antibody-dominated subtype-specific protection against influenza viruses, need to be updated and produced every year before each influenza season (fall to winter period in the northern hemisphere) because of mismatches between the vaccine strains and the prevalent circulating virus subtypes.

The adult human population has some levels of cross-protective antibodies against circulating seasonal influenza strains due to previous virus exposures or vaccinations, therefore, developing only mild disease symptoms upon infection (34, 35). By contrast, the absence of preexisting immunity to an emerging pandemic strain can lead to severe pulmonary infections and death. Infants are particularly at risk as they are immunologically naïve to influenza and may develop serious symptoms even from seasonal strains, as soon as the protection from maternal antibody wanes.

Adjuvants are formulated with the antigenic component of the vaccine to increase immunogenicity. Seasonal and pandemic influenza vaccines adjuvanted with oil-in-water emulsions AS03 or MF59, in comparison with non-adjuvanted vaccines, elicit higher titers of neutralizing antibodies with higher affinities and broader cross-reactive specificities to other influenza types and promote the persistence of long-lasting memory B cells in subjects from varied age groups (36–39).

Compared with existing cell-derived and recombinant influenza vaccines, conventional egg-based influenza vaccines are usually effective and have some limitations: a complex and slow production system, limitations in production capacity (production of single dose needed two eggs), allergic responses to egg components in some individuals, and weak cross-reactivity to other influenza subtypes (40–43). Studies have shown that cultivation of influenza virus in fertilized chicken eggs often results in adapting mutations in HA and that can alter virus antigenicity and may sometimes decrease efficacy of influenza vaccines (44–46). Mammalian cell-derived influenza vaccine provide several advantages over egg-based vaccines including influenza virus propagated in mammalian cell culture system remain unchanged, provide better or comparable protection, fast production and does not require extensive advance planning, availability of controlled production system involving bio-reactors, higher yield, faster production cycle and production can be easily scaled up by adding bioreactors (43, 47, 48). A recent study has shown comparable yield of virus from 1,000-l bioreactor, 12,000 roller bottles or 30,800 chicken eggs (47). These collective advantages increase cost-effectiveness of cell-based influenza vaccines. Despite several benefits, cell culture system also have some limitations including, scaling-up different cell lines is biggest challenge, obligation of expensive new facilities and extensive adventitious virus testing required (43, 49). These collective constraints call for new design and development of unique universal influenza vaccines that could provide long-lasting immunity against all subtypes of influenza viruses, and significantly reduce the disease burden associated with influenza-virus infections.

## Properties of Target Antigens for Vaccine Development

Antibodies that neutralize influenza-virus entry into host epithelial cells typically target HA, the main surface glycoprotein of the virion. HA is composed of globular and stalk domains and is expressed as a trimer at the surface of the virion. At the initial stage of infection, when influenza virus enters the respiratory tract, HA binds to sialic acid residues present on the surface of epithelial cells and allows the virus to be engulfed by the cells (50). The globular domain is highly variable across subtypes, whereas the stalk domain is much more conserved. Hence, the stalk domain is a potential target for the development of a universal influenza vaccine (51, 52). However, HA-head-specific antibodies have a greater neutralizing capacity than cross-reactive HA-stalk-specific antibodies, which are found at very low frequency (53, 54).

Neuraminidase is a tetrameric glycoprotein present on the surface of influenza viruses (55, 56). NA is important at the pre-infection, post-infection, and re-infections stages. NA is involved in the release of newly produced viruses from host cells and prevents the aggregation of virus particles by cleavage of sialic acids from respiratory tract mucins (57–59). NA may also participate in the fusion of viral and cell membranes (60) and facilitates budding of new virions by restricting their aggregation (60). NA is generally less immunogenic and lacking monodominant properties and therefore a less attractive target than HA (61). Despite this, NA has been used as a vaccine candidate in various vaccine formulations and as a target of various antiviral drugs, because of a lower rate of antigenic drift than HA (57, 59, 62–65).

Apart from HA and NA surface glycoproteins, the matrix proteins 1 and 2 (M1 and M2, respectively), are encoded by influenza-virus genes with partially overlapping reading frames (66, 67). M1 is a major constituent of the viral capsids and M2 functions as a proton channel in viral envelope. Studies suggest that the amino acid sequence of M2 extracellular domain (M2e) is conserved in influenza A viruses (IAV) and is, therefore, a target for the development of a universal influenza vaccine (68, 69). M2e-containing vaccines have shown encouraging results in animal models (68–71). Antibody responses to M2e protein provide protection by (1) binding to virus-infected cells and mediating effectors functions by antibody-dependent cell cytotoxicity, (2) killing infected cells by activating a complement cascade or by recruiting cells of the innate immune system, (3) preventing the release of virus particles by binding to the cell surface, and (4) phagocytosis of viral particles *via* Fcγ receptors (66, 70, 71).

Nucleoprotein (NP) is an influenza-virus protein that is associates with viral RNA and essential for viral assembly (3). NP is highly conserved across influenza subtypes and is a potential antigen for a universal influenza vaccine (72). After infection, NP is the main antigen recognized by influenza-specific cytotoxic CD8^+^ T lymphocytes (CTLs) that have the potential to kill virus-infected cells (73).

## Virus-Like Particles (VLPs)

**Figure 1:**
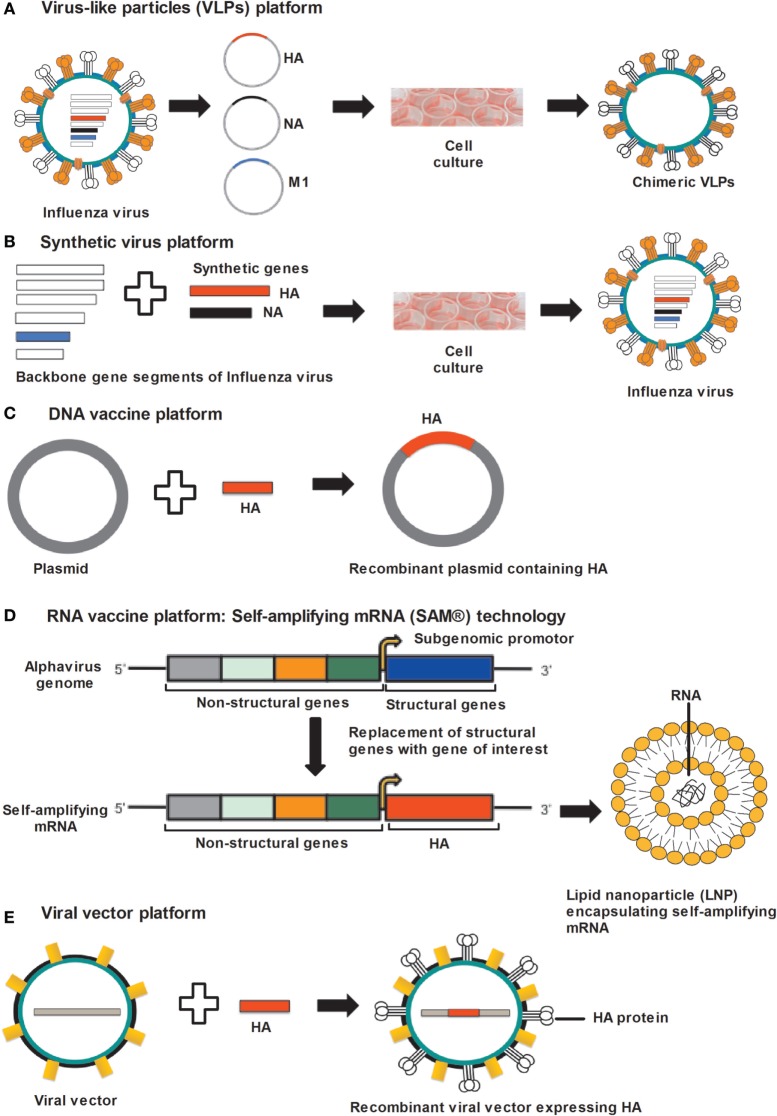
Approaches for universal influenza vaccine development. **(A)** VLP platform: VLPs produced by cloning of HA, NA, and M1 gene sequences of influenza virus into the expression vector followed by transfection in to insect cells. Co-expression of HA, NA, and M1 proteins allows self-assembly of VLPs. **(B)** Synthetic-virus platform: MDCK cells are transfected with plasmid DNA encoding influenza-virus backbone genes and error-free HA and NA gene segments, synthesized by an enzymatic and cell-free assembly technique. After transfection, vaccine viruses are rescued from MDCK cells. **(C)** DNA-vaccine platform: influenza genes encoding antigenic proteins are inserted into a DNA plasmid and delivered to the host cells, where DNA vaccines express antigenic protein. **(D)** RNA-vaccine platform: self-amplifying RNA expressed from an alphavirus genome in which structural genes are replaced by genes supporting the amplification of the RNA and the gene encoding the antigen. Self-amplifying mRNA (SAM) composed of a 5′ cap, genes encoding non-structural genes (NSP 1–4), a subgenomic promoter, the antigen-encoding gene, and a 3′ poly(A) tail. Diagrammatic representation of a lipid nanoparticle (LNP) encapsulating SAM. **(E)** Viral-vector platform: viral vector-based influenza vaccine uses a non-influenza “carrier” virus to express antigenic protein. Influenza genes encoding HA protein are placed in to the carrier virus vector to express HA protein on the virus surface. Abbreviations: HA, hemagglutinin; NA, neuraminidase; M1, matrix protein 1; VLP, virus-like particle; MDCK, Madin–Darby kidney cells.

Virus-like particles are non-infectious particles of protein multimers and are devoid of genetic material (Figure 1A). VLPs mimic certain aspects of the conformational, structural and antigenic properties of native influenza viruses, making VLPs a useful platform for vaccine development (74, 75). The development of certain universal influenza vaccines have utilized the VLP platform because VLPs have self-assembly properties, VLPs can comprise more than one protein or protein chimeras (cVLPs) and produced in a heterologous expression system, and VLPs can be formulated with adjuvants (74, 76). VLPs activate innate immunity by stimulating antigen presenting cells and this can lead to the induction of effective B- and T-cell immunity specific to those antigens present in the VLP (75, 77–79). It is reported that highly organized and repetitive protein epitopes can directly activate B-cells by cross-linking B-cell receptors (80). Furthermore, VLPs have also been shown to provoke CD8^+^ T-cells mediated immune responses through cross-presentation mechanism (81).

Virus-like particles have been used in strategies for presenting epitopes from numerous different virus subtypes and engineered epitopes derived from conserved regions of viral proteins (82–85). Most VLP-based vaccines are formulated with individual VLPs containing HA, NA, or matrix proteins, or a combination of these proteins (82, 86, 87). However, VLPs containing both HA and NA tend to predominantly induce HA-specific antibodies (41) due to the characteristic immunodominance associated with HA (88). In a recent mouse study, Schwartzman et al. evaluated a vaccine consisting of a mixture of HA-derived VLPs, from influenza subtypes H1 (H1N1; A/South Carolina/1/1918), H3 (H3N8; A/pintail/Ohio/339/1987), H5 (H5N1; A/mallard/Maryland/802/2007), and H7 (H7N3; A/Environment/Maryland/261/2006) (82). After intranasal vaccination, mice were protected against lethal influenza-virus challenge from homologous (same strains as in the vaccine; H1N1 and H7N1), intrasubtypic (antigenically different strains of the same subtypes; H5N1 and H7N9), and heterosubtypic (subtypes not included in the vaccine; H2N1, H6N1, H10N1, and H11N1) (82). Hence these results suggested that this approach was suitable for developing an effective clinical vaccine against currently circulating influenza strains and potential pandemic influenza strains. In other studies, the highly conserved HA-stalk domain was used in VLPs to induce broadly cross-reactive protective immunity (84, 88, 89). In one study, VLP formation was aided by co-expression of the HA-stalk domain with the HIV gag protein in mammalian cells (84). VLPs composed of M1 and NA from A/Puerto Rico/8/1934 (H1N1) induced cross-reactive antibody responses to an H3N2 subtype and substantial levels of protection against homologous and heterologous A subtypes, although loss of body weight was observed after lethal challenge with H3N2 (65).

Although viral-surface-protein-based VLPs have appeared promising tool for developing universal vaccine, some of the strategies have also included M2e protein. M2e is a very promising target for universal vaccine development because of the conserved nature of the protein. M2e has been included in VLPs as a genetically engineered fusion protein (e.g., with the hepatitis B virus core particle (HBc), phage derived VLPs, or with human papillomavirus L1), and vaccines from these VLPs have been shown to induce high levels of M2e-specific antibodies in mice (69, 90, 91). M2e has also been included in VLPs as a tandem repeat of five M2e variants from human, swine and avian IAV (the M2e5x vaccine) (85, 92). In mice, M2e5x protected against a lethal challenge from distinct IAV (H3N2 or H5N1) (92). Finally, in a recent study, a vaccine containing a combination of three M2e, NP, and HBc VLPs induced potent humoral and cell-mediated immunity (5).

Although the abovementioned VLP strategies have shown promise in animal models, some of the vaccine formulations have required adjuvants to enhance VLP immunogenicity. Some of these adjuvants have been based on toll-like receptor (TLR) ligands. TLRs are structurally conserved molecules that recognize ligands of microbial origins. Engagement of TLRs on innate cells results in the production of pro-inflammatory cytokines and chemokines, and an enhanced ability to eliminate the pathogens (93). Wang et al. developed chimeric VLPs containing A/Puerto Rico/8/1934 influenza virus HA and M1 and a modified flagellin protein of *Salmonella* that engages TLR5 (94). Compared with VLPs containing only HA and M1 protein, cVLPs containing TLR5 ligand flagellin was more immunogenic and protected 67% mice against lethal challenge with a heterosubtypic A/Philippines (H3N2) influenza-virus strain (94).

The advantages of VLP-based vaccines are that the immune system of the host recognizes VLPs in a similar way to the original virus particles, and chimeric VLPs induce highly effective cross-reactive heterosubtypic immune responses (94). The existence of several licensed prophylactic VLP-based vaccines (e.g., for HBV and for HPV) (95), further suggest that a VLP-based approach is a promising approach for the development of a universal influenza vaccine.

## Broadly Reactive Antibody- and T-Cell-Inducing Strategies

Antibody- and T-cell-mediated immune responses are the keys components of adaptive immune system. B cells are the source of antibodies and antibody-based immunotherapy is a very efficient approach in the treatment of various diseases caused by microbial infections (96). Most current seasonal and pandemic influenza vaccines induce high-titers of functional and neutralizing antibodies against HA and NA proteins.

Because the HA-head domain tends to contain epitopes that are immunodominant over those in the stalk, Krammer et al. investigated a strategy in which three chimeric antigens comprising a stalk domain from H1 fused with a head domain from three different subtypes, respectively, were injected sequentially in mice (97). The first vaccination with cH6/1 HA (combination of H6 head and H1 stalk) induced a very week response against the head and stalk domains, whereas a second vaccination with cH5/1 HA (combination of H5 head and H1 stalk) boosted the antibody responses against the stalk domain and induced only primary responses against the head domain (97, 98). A third vaccination with cH8/1 HA (combination of H8 head and H1 stalk) again boosted the intensity of the immune response against the stalk domain and induced a primary response against the head domain. Repeated vaccinations with chimeric HA constructs containing the same stalk domain but different heads, induced stalk-directed antibodies that mediated heterotypic immunity (98). These initial results with IAV group 1 stalk chimeric HA vaccines have since been replicated with IAV group 2 and B chimeric HA vaccines (99, 100). In addition, an inactivated split-virion vaccine adjuvanted with AS03 derived from a recombinant virus expressing an IAV group 1 chimeric HA has been evaluated in mice and shown to induce protective H1 stalk-reactive antibodies (101). Ferrets are excellent experimental animal models for the investigation of influenza-virus pathogenicity and immunobiology because of their susceptibility to influenza-virus infection and ability to develop disease symptoms similar to humans (102–104). Sequential immunization studies with chimeric HA (generated by exchanging head domains and retaining same HA-stalk domain), successfully induced broadly reactive antibody responses in ferrets (104–106).

An alternative strategy based on broadly reactive monoclonal antibodies could confer cross-protective and long-lasting immunity against influenza-virus infections. Studies on monoclonal antibodies have shown that conserved regions exist within the HA head domain. Whittle et al. isolated a human monoclonal antibody, CH65, directed against the globular head region that was able to neutralize the infectivity of about 30 H1N1 strains (107). Lee et al. demonstrated that an antibody fragment, derived from the S139/1 monoclonal IgG antibody, targets highly conserved residues in the receptor binding site of the head domain. This antibody fragment neutralized H1N1 and H3N2 strains. However, the parental antibody neutralized many more strains from H1, H2, H13, and H16 subtypes suggesting that the avidity associated with bivalent interactions of the antibody and HA molecules contributed to enhancing cross-reactivity (108).

As discussed earlier, the stalk region of HA is highly conserved and stalk-specific neutralizing antibodies cross-react with different virus subtypes. Okuno et al. isolated a monoclonal antibody, C179, from A/Okuda/1957 (H2N2)-immunized mice (51). C179 inhibited the fusion activity of HA and neutralized influenza subtypes H1, H2, H5, and H6 (51, 52). Throsby et al. isolated a monoclonal antibody, CR6261, from human IgM^+^ memory B cells that neutralized antigenically diverse H1, H5, H6, H8, and H9 influenza subtypes (109). Corti et al. isolated a monoclonal antibody, F16, by screening human plasma cells from peripheral blood. This monoclonal antibody was able to recognize HA of all 16 subtypes of influenza virus and efficiently neutralized groups 1 and 2 viruses (110). Recently, Dreyfus et al. isolated from a phage display library, a monoclonal antibody—CR9114—that recognized the HA-stalk domain of antigenically distinct influenza A and B viruses (111). This antibody protected against lethal challenge from both influenza A and B viruses (111). In most of the broadly cross-reactive stalk antibodies identified, the VH1-69 gene of the heavy-chain variable region is responsible for binding to the HA stalk. Recently, Pappas et al. identified the pathway for the generation of VH1-69 antibody and developed broadly neutralizing antibodies by mutation (112).

Innovative approaches based on optimal antigen design, have been implemented for the induction of cross-protective antibodies. In these approaches, antigens contain only cross-protective epitopes. Recently, Giles et al. designed vaccine antigens based on HA of H5N1 subtype by a computational approach, computational optimized broadly reactive antigen, and these antigens induced broadly reactive antibodies in mice and ferrets after challenge with H5N1 (113, 114). In another approach, mutations were incorporated in HA2 (HA1 and HA2 are subunits of HA, enzymatic cleavage product of HA0 precursor protein) of HA antigen and expressed in *Escherichia coli*. The resulting protein was highly immunogenic and protected mice against a lethal challenge by homologous and intrasubtypic viral strains (115).

These promising studies may be helpful for the development of an antibody-based first line of defense in the case of an emerging pandemic.

Historically, the induction of neutralizing antibodies has been the main focus in the development of influenza vaccines. However, recent data suggest that the induction of T cells with cross-reactive properties is highly important (116–118). These T cells are accountable for providing heterosubtypic immunity, wherein memory T cells generated against conserved epitopes of previous influenza virus can cross-react against multiple influenza subtypes. Both CD4^+^ and CD8^+^ T cells induce heterotypic immunity against internal and surface proteins (119–125). CTLs are the main effector cells that take part in antiviral immunity. Numerous studies have shown that CTLs orchestrate heterosubtypic effector functions against influenza virus infected cells by direct cytotoxic inhibitory mechanisms (101–105). A recent study by Sridhar et al. demonstrated a significant role of cross-protective CD8^+^ T cells, specific for conserved core protein epitopes, in individuals lacking cross-neutralizing antibodies (126). A significant positive correlation was found between higher frequencies of preexisting CD8^+^ T cells to conserved epitopes and the low grade of clinical illness among healthy individuals lacking protective antibodies against pH1N1 virus (126). These observations may help in the development of a universal influenza vaccine either by improving currently available vaccines or by designing T-cell epitopes targeting all subtypes of influenza viruses (126).

CD8^+^ T lymphocytes specific for conserved influenza epitopes has been identified in several studies (121–123). Kreijtz et al. showed that in humans, CTLs specific for H3N2 influenza virus display cross-reactivity with H5N1 influenza virus suggesting that a certain number of common epitopes are present in both viruses (121). Furthermore, liposomes containing full-length recombinant H5N1 NP and M2 antigens induced CTLs and showed high efficacy in mice (122). Another promising adjuvant is ISCOMATRIX (IMX), which immunomodulates humoral and cellular immune responses by inducing cytokines, enhancing antigen presentation by dendritic cells, inducing innate immune responses and cross-presentation of exogenous antigens to CD8^+^ T cells (123, 127, 128).

The role of CD4^+^ T-cells in mediating heterosubtypic immunity is less well understood. CD4^+^ T cells confer cross-protection by cytolytic functions (perforin-mediated) or helping B or T cells (129, 130). It has been shown that M1- and NP-specific memory CD4^+^ and CD8^+^ T cells generated by seasonal influenza A virus infection in humans could cross-react with an avian H5N1 strain (120). HA2 (stem domain) subunit is very conserved across the different influenza HA subtypes and also a promising vaccine candidate (131, 132). Some cross-reactive CD4^+^ T cell epitopes have been identified in this subunit (124, 131). Lee at al generated a recombinant HA2 polypeptide from influenza A/EM/Korea/W149/06 (H5N1) and reported homologous and heterologous CD4^+^ T cell responses in mice, immunized with HA2 protein (132).

As published reports suggested that the majority of T cell-based vaccines focus on conserved T-cell epitopes in NP and M proteins and are helpful in reducing disease progression and mortality against heterologous virus infections (133, 134). Furthermore, T-cell inducing approaches are unable to prevent infections. Therefore, a combination of antibody and CTL-inducing strategies may be required for an effective universal influenza vaccine development (133). Ongoing clinical trials based on coadministration of seasonal influenza vaccine with MVA-NP + M1 (viral vectored vaccine based on modified vaccinia virus Ankara expressing influenza NP and M1 proteins) and prime-boost strategies induced both T-cells and antibody responses (133, 135, 136).

## Synthetic Influenza-Virus Platform

A recent technology platform that utilizes *reverse genetics* technology for the development of synthetic vaccine viruses was set up after the 2009 H1N1 pandemic (137). Dormitzer et al. developed an error-free gene assembly system for the synthesis of influenza-virus vaccine strains (137). In the first step, accurate and error-free HA and NA gene sequences are synthesized with the help of an enzymatic cell-free gene assembly technique with enzymatic error correction, followed by transfection in to the Madin–Darby canine kidney (MDCK) cells along with plasmid DNA coding viral backbone genes (137) (Figure 1B). In the final step, viruses for use in vaccines were rescued from MDCK cells. It was possible with this method to maintain HA and NA sequences identical to the original viruses (137, 138). As a proof-of-concept, artificially synthesized and wild-type H7N9 viruses were used and showed comparable immunogenicity (137). This breakthrough technology avoids the use of millions of eggs needed for virus propagation and prevents the mutations usually occurring during this step. Presently, the synthetic-virus technology is limited to the preparation of viruses for vaccine manufacturing (139). However, by applying this technology, error-free universal vaccines could be produced in a very short period of time in the future.

## Nucleic Acid Platform

The feasibility of developing vaccines based on nucleic acids has been investigated for a long time. Nucleic acid vaccines have attributes for being safe and effective, and combine the positive attributes of live-attenuated and subunit vaccines. Nucleic acid vaccines are composed of DNA or RNA sequences encoding the antigen. These vaccines are delivered by viral particles competent for entry in host cell, by formulation with lipids or emulsions, or by means of electroporation (140).

### DNA Platform

DNA vaccination is a technology developed about two decades ago (119–121) (Figure 1C). The ease of production in a very short period of time, stability and a very cost-effective production process makes this technology attractive for vaccine development (141). DNA vaccines are non-infectious, non-replicating, and do not induce vector-specific immunity (141). However, no DNA vaccine has been approved for human use to date.

DNA vaccines are delivered by various methods including the gene-gun approach and electroporation. The gene-gun approach involves the intracellular delivery of DNA in epidermal cells by bombarding the skin with DNA coated gold particles (142). This approach has not been explored for human use. In the electroporation method, DNA molecules are delivered by combined electric pulses that transiently permeabilize the cell membrane and promote DNA movement toward the membrane (143). Efforts are ongoing to deliver DNA vaccines efficiently, e.g., needle-free delivery and mucosal delivery (144, 145). Although DNA vaccines induce potent immune responses in animal models, they have appeared ineffective in humans because of suboptimal immunogenicity (145). There are other drawbacks of DNA vaccines including the degradation of DNA by host enzymes, the requirement for the DNA to enter the nucleus, and the possible risk of DNA integration into the vaccine recipient’s DNA (145).

The immunogenicity of various influenza virus DNA-vaccine candidates has been assessed in animal and human models (146–148). Smith et al. conducted a phase 1 study with an H5 HA-based DNA vaccine adjuvanted with Vaxfectin, and the results showed protective responses to the vaccine in about 67% of the recipients (147). A polyvalent DNA vaccine, also expressing H5 HA antigens (genes from H5N1 viruses A/Hongkong/156/97, A/Vietnam/1203/04, A/Anhui/1/2005, and A/Indonesia/5/2005) has been evaluated in rabbits and induced cross-protective antibodies against different clades (149). Results from this study in mice suggested that plasmid DNAs encoding NA from H3N2 virus provided cross-protection against lethal challenge with antigenically different viruses within the same subtype, but not against a different, H1N1, subtype (150). DNA vaccines expressing conserved NP and M1 antigens, or the expression of fusion proteins (HA and NA; or NP and M1) have been evaluated in mouse models of influenza-challenge (122, 128, 129). These strategies conferred broad protection against homologous and drifted viruses (151).

Although DNA is a flexible platform that has shown cross-protective efficacy in preclinical challenge models, it has yet to show potency in human clinical trials (152). Improvements are in progress for a broadly protective vaccine including the implementation of heterologous prime/boost strategies, where the second encounter with the heterologous influenza strain (boost) also induces cross-protective immunity by expansion of a preexisting pool of memory cells against conserved epitopes of the viral proteins (153). In an experimental setup, the sequential immunization of BALB/c mice with DNA coding for HA from H3N2 viruses arising approximately 10 years apart (prime: A/Hong Kong/1/1968, A/Alabama/1/1981 and A/Beijing/47/1992, and boost A/Wyoming/3/2003) elicited the production of antibodies with broad cross-reactivity against multiple H3N2 viruses (34).

### RNA Platform

RNA-based vaccines are the most recent version of nucleic acid-based vaccines and possesses several benefits over DNA vaccines. In 1990, Wolff et al. (154) demonstrated that the direct injection of messenger RNA (mRNA) intramuscularly resulted in the expression of the encoded protein in a mouse model. In contrast to DNA vaccines, which function by DNA entering the nucleus of the vaccine recipient’s cell, mRNA vaccines function by the translation of mRNA in the cytoplasm (140, 155). Currently, there are two kinds of mRNA-based vaccines being developed: (1) conventional non-amplifying mRNA and (2) self-amplifying mRNA (SAM)-based vaccines (156).

The ease of construction, the small size (about 2–3 kb), and the absence of additional protein-encoded sequences are qualities of non-amplifying mRNA vaccines, although a shorter half-life and low protein expression make non-amplifying mRNA less attractive over SAM vaccines (157). However, nucleotide optimization may increase the potency and stability of the non-amplifying mRNA vaccines (158). Efficient delivery of mRNA in to the cytoplasm of the cell is the key challenge. Various delivery technologies have been explored for conventional non-amplifying mRNA, including administration of naked mRNA by injection through different routes (intradermal, intranodal, subcutaneous, and intramuscular) (159–162), or administration mRNA premixed with liposomes or lipopolyplexes (163, 164). Lipid–mRNA complexes have been shown to enhance the immunogenicity of various mRNA vaccines (165, 166). Preclinical and clinical studies suggest that non-amplifying mRNA vaccines are effective in mice, non-human primates and humans (159, 167). Recently, Moderna Therapeutics published the results of a phase 1 clinical trial study of a modified mRNA vaccine encoding HA protein of avian H10N8 formulated in lipid nanoparticles (LNPs) and demonstrated robust immunogenicity in humans (167). Furthermore, an HA-encoded mRNA vaccine conferred protection in new born and old BALB/c mice (18 months of age), suggesting that mRNA vaccine are capable of reducing influenza disease burden at the extremes of age (159). This latter aspect is relevant because the disease burden of influenza virus is greater in children and the elderly. Cross-protective T-cell responses to heterologous virus strains were also evident when mice were immunized with an NP-encoded mRNA vaccine. The combination of multiple antigens may be more effective to induce broadly protective immune responses, as reported with VLP-based vaccines.

Self-amplifying mRNA vaccines of sizes around 9–10 kb, have been generated using alphavirus genome as a vector, where alphavirus genes encoding structural proteins were replaced by the gene(s) of interest, therefore disabling the production of infectious viral particles (157, 168) (Figure 1D). Delivery of mRNA into the cell cytoplasm allows the translation of open reading frames encoding for non-structural proteins (nsP 1–4), which function as RNA-dependent RNA polymerase (RDRP) and produce a negative-sense copy of the genome (157). With the help of RDRP, this negative-sense RNA serves as template for positive-strand genomic mRNA and shorter subgenomic mRNA. The subgenomic mRNA is transcribed at a very high copy number resulting in high level of protein-antigen expression. The auto-amplification properties of SAM vaccines allow high-level protein expression of the vaccine antigen(s).

The effective delivery of mRNA vaccines, within acceptable limits of tolerability, is a key aspect for improving the vaccine efficacy. LNPs and cationic nano-emulsions synthetic non-viral delivery systems were used successfully with SAM in recent studies (103, 168–171). Geall et al. evaluated a synthetic LNP formulation of SAM as a way to increase the efficiency of antigen production and immunogenicity *in vivo* without the need of a competent viral delivery system (169). This novel vaccine technology was found to elicit broad, potent and protective immunity that was comparable to viral delivery, but without the inherent limitations of viral vectors.

Self-amplifying mRNA (HA) vaccines induce potent functional immune responses in mice and ferrets, comparable to those elicited by a licensed influenza subunit vaccine preparation (168). In mice, SAM (HA) derived from influenza A/California/7/2009 (H1N1) strain protected from a lethal challenge with a heterologous influenza strain A/Puerto Rico/8/1934 (H1N1) (103). This cross-protection may have been mediated by CD4^+^ and CD8^+^ T cells, because A/Puerto Rico/8/1934-specific CD4^+^ and CD8^+^ T cells were induced by the vaccine but not A/Puerto Rico/8/1934-specific antibodies (103). Recently, SAM vaccines encoding NP and M1 antigens, separately or in combination, delivered with LNP induced CTLs in mice (170). The expansion of central memory and effector memory CD4^+^ and CD8^+^ T-cell populations was also observed. The coadministration of monovalent inactivated influenza vaccine (MIIV) along with SAM vaccine also enhanced MIIV efficacy against heterologous challenge and enhanced the frequency of HA-specific T-cells (170).

In epidemics or disease outbreaks, the rapid development of vaccines is essential, and this is potentially feasible with SAM technology. Within 7 days, SAM (HA) vaccine against H7N9 (A/Shanghai/2/2013) was developed (168). The SAM vaccine technology is still in a preclinical development; nevertheless, present data suggest that the SAM technology has the potential to provide a platform for rapid and cost-effective vaccines.

### Viral Vectors

Viral vectors with the capability of replication within the cells of the vaccine recipient are being evaluated as vaccines in the form of viral delivery of nucleic acids encoding the gene of interest (Figure 1E). The viral vector is non-infectious for the host but can express the antigen over a certain period of time.

Viral-vector-based influenza vaccines have been evaluated using double-stranded DNA (adenovirus and poxvirus) and single-stranded RNA (alphavirus, parainfluenza virus 5) viruses (172–175). Recently, by exploiting MVA vector and *in silico* mosaic approach, a recombinant vaccine construct, MVA-H5M (HA of H5N1), was developed (176). Mosaic technology allows the selection of potential epitopes by using a genetic algorithm (177). Immunization of BALB/c mice, MVA-H5M induced broadly protective neutralizing antibodies against clade 0–2 (avian influenza) and highly pathogenic A/Puerto Rico/8/1934 (H1N1) virus and induced cellular immunity (176). An adenovirus vector expressing NP and M2 proteins conferred protection from highly virulent H5N1, H1N1, and H3N2 influenza viruses (178) as well as reduced viral transmission from vaccinated and infected mice to unvaccinated mice (178, 179). Furthermore, Kim et al. employed a novel approach to inducing cross-reactive immune responses against HA protein by generating recombinant human adenovirus 5 vector encoding full length HA (from H5N1) and four tendem copies of M2e domain (rAdH5/M2e) (180). Adenovirus vaccine induced antibody-mediated cross-protection against heterosubtypic viruses in mice (180).

Viral-vector-based vaccines possess several advantages including the capability of inducing both humoral- and cell-mediated immunity, a high level of protein expression and long-term stability (181–184). Some of these viral-vector systems can mimic aspects of natural influenza-virus infection because of their direct delivery to the mucosa (3). Vector-based vaccines also possess several limitations including reduced immunogenicity in the presence of preexisting immunity (185) and risks of pathogenesis in certain individuals (186).

## Conclusion

An optimal universal influenza vaccine should provide cross-protection against all subtypes of influenza viruses, particularly historic and circulating strains. Many of the universal influenza vaccines in development target conserved domains of surface and internal proteins. Numerous approaches are being applied for the development of universal flu vaccines. Proof-of-concept and initial results support the promise of these approaches (168, 187). Novel platforms focused on influenza vaccine development are currently in preclinical and early clinical development (Table 1). Applying these novel platforms may offer additional benefits for the development of a universal influenza vaccine.

**Table 1:** Current development stage of the influenza vaccines based on novel platforms.

Platform	Development stage					Reference
		
	Preclinical	Phase 1	Phase 2	Phase 3	Licensed	
Virus-like particles	✓	✓	✓	⨯	⨯	(188–190)
Broadly reactive antibodies and T-cell inducing strategies	✓	✓	✓	⨯	⨯	(136, 191–194)
Synthetic virus	✓	⨯	⨯	⨯	⨯	(137)

**Nucleic acid**						
• DNA	✓	✓	⨯	⨯	⨯	(147, 195, 196)
• RNA	✓	✓	⨯	⨯	⨯	(103, 167, 170)
• Viral vector	✓	✓	✓	⨯	⨯	(134, 197, 198)

## Author Contributions

All authors were involved in drafting the manuscript and revising it critically for important intellectual content.

## Conflict of Interest Statement

Presently, SB is an employee of the GSK group of companies while AK and TSM were employed at the time of study conduct. AK and TSM are now employed with Linköping University, Sweden and Technical University of Denmark, Denmark, respectively. All the authors declare that they have no other conflicts.
